# Hourly weather observations from the Scottish Highlands (1883–1904) rescued by volunteer citizen scientists

**DOI:** 10.1002/gdj3.79

**Published:** 2019-08-26

**Authors:** Ed Hawkins, Stephen Burt, Philip Brohan, Michael Lockwood, Harriett Richardson, Marjory Roy, Simon Thomas

**Affiliations:** ^1^ National Centre for Atmospheric Science, Department of Meteorology University of Reading Reading UK; ^2^ Department of Meteorology University of Reading Reading UK; ^3^ Met Office Hadley Centre Exeter UK; ^4^ National Centre for Atmospheric Science University of Leeds Leeds UK; ^5^ Scottish Centre Royal Meteorological Society Edinburgh UK

**Keywords:** atmospheric science, Ben Nevis, citizen science, climate, Fort William, mountain climate, Scotland, weather

## Abstract

Weather observations taken every hour during the years 1883–1904 on the summit of Ben Nevis (1345 m above sea level) and in the town of Fort William in the Scottish Highlands have been transcribed from the original publications into digital form. More than 3,500 citizen scientist volunteers completed the digitization in less than 3 months using the http://WeatherRescue.org website. Over 1.5 million observations of atmospheric pressure, wet‐ and dry‐bulb temperatures, precipitation and wind speed were recovered. These data have been quality controlled and are now made openly available, including hourly values of relative humidity derived from the digitized dry‐ and wet‐bulb temperatures using modern hygrometric algorithms. These observations are one of the most detailed weather data collections available for anywhere in the UK in the Victorian era. In addition, 374 observations of aurora borealis seen by the meteorologists from the summit of Ben Nevis have been catalogued and this has improved the auroral record for studies of space weather.

## 1 INTRODUCTION

Between December 1883 and September 1904, a group of meteorologists undertook detailed weather observations at the summit of Ben Nevis, the highest mountain in the United Kingdom, at 1345 m above sea level. Every hour during the day and night, one of the meteorologists recorded detailed observations of the weather including atmospheric pressure, temperature (both wet‐ and dry‐bulb), precipitation, wind strength and direction, sunshine and cloudiness. They also made detailed notes of atmospheric phenomena such as aurorae, haloes and glories.

**Figure 1:**
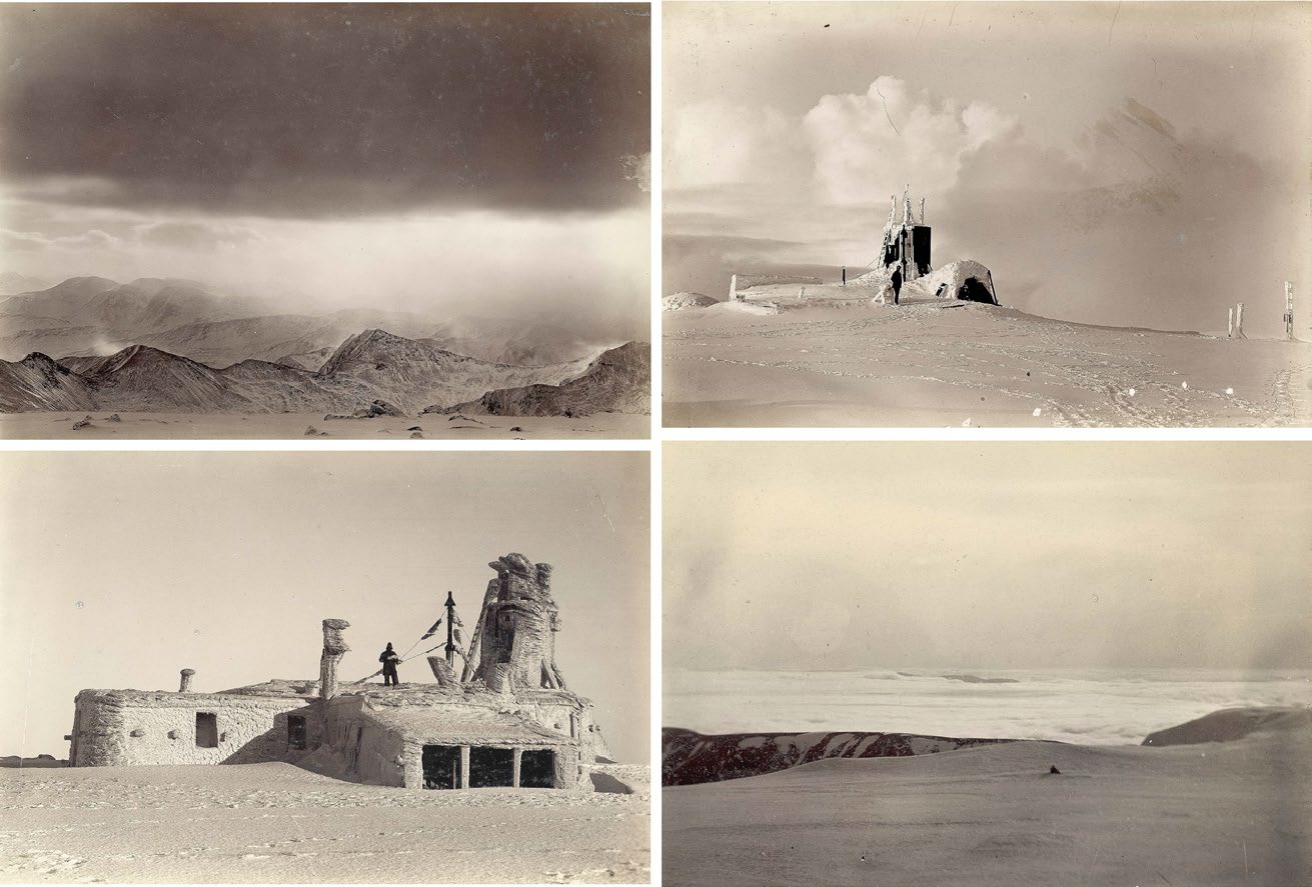
Photographs taken on the summit of Ben Nevis by the Victorian era meteorologists. Images from Royal Meteorological Society collection, held as part of the Met Office archive at National Records of Scotland

Weather conditions in the winter could be so severe that it would not be possible for anyone to make the climb to the observatory. Supplies had to be brought up by pony between June and November. Kilgour (1905) and Roy (2004) provide detailed accounts of life at the observatory. Some photographs taken by the observers at the summit are shown in Figure 1.

Between August 1890 and September 1904, similar weather observations were taken near sea level in a dedicated observatory in the town of Fort William, just a few kilometres from the summit of Ben Nevis. The same hourly weather observations were recorded, except that the cloudiness and wind measurements were not taken. Between December 1883 and December 1890, observations were also taken at Fort William School several times daily.

The observatories were set up and run by the Scottish Meteorological Society. A public appeal was launched in 1883 to fund the summit observatory which was so successful that the project was able to go ahead that year. There were a number of sources of income, including from the Meteorological Office for copies of the Ben Nevis and Fort William observations. Various substantial donations were received from time to time, including from Queen Victoria, and regular small donations were received from the public. The permanent staff (the superintendent, two or more assistants and the cook) were paid a modest salary, and there were also volunteers who occasionally relieved the permanent staff. With the lack of substantial government funding, the summit observatory was in financial difficulty for much of its period of operation. It finally had to close in October 1904 because the annual cost of running it was far larger than the income received.

The observations were all published in the *Transactions of the Royal Society of Edinburgh* in four volumes (Buchan, 1890; Buchan and Omond, 1902, 1905, 1910) but they have never been digitized. These data arguably represent the most detailed set of weather observations for this period anywhere in the UK and certainly in a montane environment. No regular weather observations have been taken on Ben Nevis since the summit observatory closed.

The transcription (or ‘rescue’) of these data from paper to digital format has now been completed with the help of thousands of volunteer ‘citizen scientists’. This paper describes the data rescue process and makes the data openly available for anyone to use. This fulfils the ambitions of the meteorologists of over a century ago that their data be made available to aid weather forecasting and the study of mountain meteorology. The volunteer approach to the data rescue is particularly fitting given that both observatories were partly crowd‐funded.

## 2 CITIZEN SCIENCE APPROACH TO COLLECTING DATA

### 2.1 Website development

**Figure 2:**
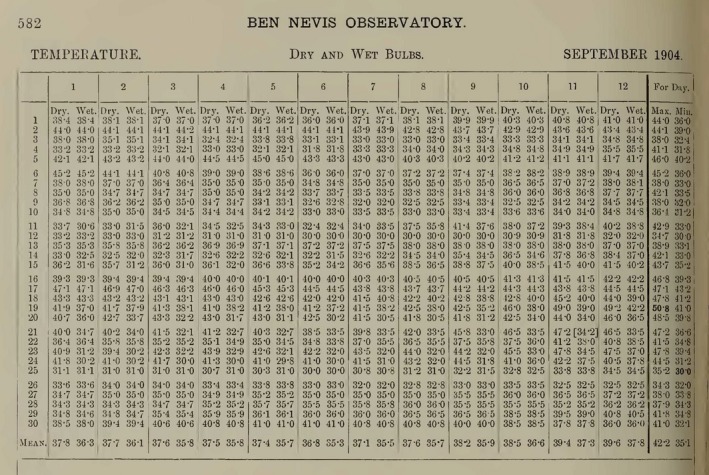
Example page from the published volumes, showing temperature observations for the Ben Nevis Observatory for the morning hours in September 1904 in degrees Fahrenheit. The columns indicate the time of day (in GMT), with sub‐columns for the dry‐ and wet‐bulb thermometer data. The rows are the day of the month, with a mean along the bottom row. The final two columns show the maximum and minimum hourly values for the dry‐bulb thermometer that day

An example page from the published volumes is shown in Figure 2, showing the temperature observations for September 1904 (in °F)—the final month of measurements. Note the additional rows and columns documenting the mean, max and min which are valuable cross‐checks for the data. In total, ~2,100 images like this were available from scans of the original published documents.

The Zooniverse (http://zooniverse.org) provides a popular way of developing citizen science projects. Using their Project Builder interface, a custom website was created to enable volunteers to transcribe the data from the images. A ‘beta’ version of the website was produced and reviewed by a selected group of experienced Zooniverse volunteers who provided valuable feedback and suggestions for improvements.

The images were split into several batches, selected by observation type and observatory. The volunteers were required to complete four tasks on a randomly selected image from a particular batch. They were asked to confirm that the image displayed was for the expected weather type and observatory, for example temperature on Ben Nevis. They were then asked to select the month and year the data were for from a set of drop‐down menus, followed by a request to draw a box around a specific column of data, for example the wet‐bulb temperature at 4 a.m. They then typed the data shown in that column, including the bottom row which represented the mean (or sum for the precipitation data). A total of around 52,000 columns were entered. Each column was keyed by at least three independent volunteers so that occasional and inevitable errors could be detected during the quality control process (see below).

Assuming it took an average of 4 min to complete the tasks for a single column, this equates to over 10,000 person‐hours of effort, or over 6 years on a full‐time basis. The website launched in September 2017 and in less than 3 months all of the image tasks had been completed with more than 3,500 volunteers completing the transcription of at least one column. Around 700 volunteers were responsible for more than 75% of the transcriptions.

### 2.2 Choices made and lessons learnt

From the start of the website design, it was considered that asking volunteers to enter an entire table (like shown in Figure 2) was too much for a single task. The challenge was to break the project into smaller, more manageable pieces. For example, a decision was needed about whether to ask for specific rows or columns of data. Due to the image dimensions, it was decided that it was easier for the volunteers to type in a whole column, rather than a row, to minimize the amount of required zooming and scrolling around the image. However, recent evidence from other data rescue efforts suggests that it is easier to make mistakes reading down a column than across a row (Ryan *et al.*, 2018).

A decision was also required about how many volunteers would be needed to type in each column to ensure accuracy. Obviously, the more repeats required, the slower progress would be, but the resulting data would require less manual correction of errors. We decided on three repeats per column. The presence of the extra tabulated rows and columns for the mean or sum also provided an extra check on the accuracy of the keying and were used to find errors (see below).

It was also decided that the project needed to be split into batches of images as we were uncertain about the number of volunteers that might be recruited. The last 7 years of the observatory data (1898–1904) was the first batch chosen, with the aim of completing the transcription of a short period at the very least. In fact, the project was far more popular than expected so the images for 1893–1897, 1888–1892 and finally 1883–1887 were added in turn. These were the four periods in the published volumes, ensuring consistency of table design within each batch.

A decision was also required about which weather variables we wanted to rescue. Brönnimann *et al.* (2006) describe a process to consider when making such decisions. Temperature, precipitation and pressure were agreed as the highest priority, although around half of the Ben Nevis wind observations were also rescued (1893–1904). The cloudiness and sunshine data, and the remaining wind observations, are still undigitized, but the images are available for anyone to examine, especially for particular case studies.

There are several factors which helped ensure the success of this project. The story of the intrepid weathermen living in such a remote environment, struggling with the weather, is a wonderful ‘hook’ to get people interested in becoming involved. As a result, we were able to get coverage of the project in the media, especially the BBC (Amos, 2017) and in Scottish local newspapers. Twitter was also a highly useful tool for spreading information about the project. In addition, actively engaging with the volunteers through the website forums and providing regular updates on their progress was essential and provided reassurance to the volunteers that they were doing useful science. Several volunteers commented that the Weather Rescue project team was one of the most engaged they had seen across a range of Zooniverse projects and this was helpful and encouraging to them.

### 2.3 Data processing and error checking

The data collected through the web interface was made available to the project team. For each task, a single string could be extracted representing the typed observations for the requested column. This string had to be converted and separated into values for individual table cells so that the entries from different volunteers could be compared. Where at least two of the three typed entries agreed, the value was provisionally accepted. If there was complete disagreement, then the uncertain value was flagged for manual checking.

The extraction of individual values from a long string was sometimes awkward, especially in the early stages of the project due to some misinterpretation of the instructions, which was quickly resolved. For later phases of the http://WeatherRescue.org project, we have implemented a different data entry system to avoid some of these issues by requiring separate input text boxes for each observation in the online interface.

The data for each variable and each month were output as individual spreadsheets, representing the digital equivalent of each page (Figure 2). First, the flagged values where the volunteers all disagreed were added manually by consulting the original documents. The spreadsheets included an extra row and column for the calculated mean (or sum for precipitation) of the transcribed values which was compared with the transcribed mean (or sum). Where the transcribed mean or sum value disagreed with the calculated value, every hourly value was checked against the original page. Usually, the source of the error was evident, but once obvious errors were corrected some calculated means remained different to the published values. Where the error was limited to the last significant digit, the problem was assumed to be rounding errors and the calculated mean used. Occasionally, the error was greater than this, and where the hourly values appeared plausible in continuity checking within the diurnal cycle and valid within meteorological parameters, the error was assumed to be within the published mean and the calculated mean was substituted, rather than make possibly arbitrary changes within the published hourly dataset. Changes to the published values were made only as a last resort, and most often these were clearly justified by a typographical error in the printed report.

The quality of the crowdsourced data was extremely high—when using three volunteers per observation, the correct value was obtained more than 95% of the time. The most frequent cause of missing or mistyped data values within a column was when identical values occurred on consecutive rows, when the eye would presumably skip to the second value and continue from there. This was particularly marked with precipitation observations, which were often zero, and for wind observations, many of which look very similar, particularly in the summer months. Errors made when transcribing temperature observations were less frequent than for pressure, perhaps because less numbers had to be typed per observation or the range of values (in °F) were more familiar; pressure readings, in inches of mercury, being less familiar to a public audience had an error rate about twice that of temperature.

After correcting for mistyped or missing transcribed data, almost all remaining errors were due to typographical or arithmetical errors in the original pages; for example, a 9 was typeset as a 6. If these errors occurred in the significant digits, then these were obvious; for example, if the pressure appeared to drop from close to 29–26 and back to 29 inches/Hg within the space of 2 hr, it was clearly physically implausible, given there were no observed tornadoes! As a testament to the standards of the original published volumes, in approximately 182,500 Ben Nevis Observatory dry‐bulb hourly temperatures (December 1883 to September 1904), 153 errors were identified, an error rate in the published pages of just 0.08%. Similarly, for Fort William pressure observations from August 1890 to September 1904, approximately 132,000 observations, 180 errors were identified, just 0.14%.

However, it is inevitable that there are additional errors made in the less significant digits which we will never discover but these will likely be within the observational uncertainties. It was also noted that the volunteers made less errors as the project progressed—they clearly became more aware of the likely ranges of the data being entered and more likely to pick up their own errors. They also discussed typographical errors they had spotted in the project discussion web pages.

## 3 ORIGINAL OBSERVATIONS AND UNIT CONVERSIONS

### 3.1 Equipment used

The equipment and observation practices were constant throughout the record. The Fort William Observatory was provided with standard automatic recording equipment by the Meteorological Office. This used continuous photographic recording of temperature and barometric pressure in a North Wall screen, with hourly values being extracted from the traces. Check readings were made several times a day to correct if necessary the scale of the traces. A self‐recording Beckley rain gauge was used to provide the hourly rainfall totals.

At the Ben Nevis Observatory, because of the severe icing which could occur during the greater part of the year, self‐recording instruments could not be used and hourly manual observations were made by the observers. Pressure readings were obtained from a Fortin mercury barometer mounted in the office. The charts from a Richard's aneroid barograph were used as a check. Dry‐ and wet‐bulb thermometers were mounted in a standard Stevenson Screen during the summer months—the ground below the screen was broken rock with no vegetation. When snow was on the ground, the thermometers were housed in screens on ladder‐like stands so that the screens could be raised or lowered to keep the thermometers between 3 and 5 feet (1–1.5 m) above the surface. Because of the icing, self‐registering maximum and minimum thermometers were not used, and if a screen became severely iced up, it was taken inside to thaw out and a substitute screen and thermometers used. When the temperature was below 0°C, great care was taken to make sure that, before the reading was made, the muslin on the wet‐bulb thermometer was coated with ice—becoming an ice bulb. During major storms, when it would have been unsafe to go out to the screen, temperature readings were obtained from thermometers mounted on the outside of the tower, whose scales could be read from inside the tower. We are not able to provide information about which observations were made like this, but the different thermometers were regularly cross‐checked and calibrated by the observers.

For the Ben Nevis observations, the daily minimum and maximum dry‐bulb temperatures are the lowest and highest hourly observed values, whereas for Fort William they were recorded using separate screened minimum and maximum thermometers.

Two duplicate rain gauges were used at the summit. They were of 5 inches (127 mm) diameter and had rounded bases so that they could be set with their top 1 foot (300 mm) above the surface and levelled. They were exchanged each hour, being brought inside for measurement of the rainfall or melting of snow. Hourly sunshine figures were obtained from the charts provided by a Campbell–Stokes sun recorder, which had an unobstructed horizon. Wind direction and force were noted by an observer standing on the roof of the observatory using a Ben Nevis scale 0–12 (see below), and consistency between observers was checked. During the summer months, comparisons were made between the force estimates and the hourly winds recorded by a Robinson cup anemometer mounted on the tower, enabling equivalence tables of wind speed to be derived and published (Table 1). Cloud species and amount (on a scale of 1–10) were recorded, and other phenomena (e.g. thunderstorms, haloes, glories, St Elmo's Fire and aurorae) were noted.

**Table 1:** Conversion from tabulated wind forces to knots, m/s and mph

Ben Nevis Force	0	1	2	3	4	5	6	7	8	9	10	11	12
Knots	0	5	10	18	26	34	43	52	63	73	84	97	113
m/s	0	2.5	5.0	9.1	13.1	17.1	21.6	26.2	31.7	36.7	42.2	48.8	56.8
mph	0	5.6	11.3	20.3	29.3	38.3	48.4	58.5	70.9	82.1	94.5	109.1	127.1

### 3.2 Additional observations made at Fort William School

Between December 1883 and December 1890, before the Fort William Observatory was opened, regular weather observations were undertaken at Fort William School (56.82°N, 5.11°W, elevation 11 m), including pressure (five times daily, reduced to 32°F and to mean sea level), dry‐ and wet‐bulb temperature (both five times daily), minimum and maximum dry‐bulb temperature, daily precipitation, wind strength and direction (twice daily) and cloudiness (three times daily). These were published in Buchan (1890) and Buchan and Omond (1902) and have also been rescued. The published format of those observations was very different from the observatory logbooks, so a separate effort from the volunteers was requested. A spreadsheet template was made available for the volunteers to download, type in a specific month of data and send back to the science team for checking.

### 3.3 Conversion factors and locations

We have converted all the pressure observations from the values tabulated to 3 decimal places in units of inches/Hg to millibars (hPa) by multiplying by 33.8639. The precipitation, measured in inches to 3 decimal places, has been converted to mm by multiplying by 25.4. Temperatures have been converted from Fahrenheit, measured to 1 decimal place, to Celsius by subtracting 32.0 and dividing by 1.8. The daily rainfall amounts, and minimum and maximum temperatures are given for the period from midnight to midnight. Equivalent estimates for other periods could be calculated from the hourly data.

The resulting pressure and temperature values have been rounded to 1 decimal place and the rainfall to 2 decimal places. The pressure observations at both Ben Nevis and Fort William had already been corrected to a temperature of 32°F, and at Fort William had already been reduced to mean sea level. The altitude of the Fort William Observatory was 42 feet or 13 m. The Ben Nevis pressure observations are not corrected to mean sea level and were taken at 1345 m above sea level. The Ben Nevis Observatory location was at 56.80°N, 5.00°W, and the Fort William Observatory was at 56.81°N, 5.12°W.

The wind speed was recorded in ‘Ben Nevis force’, which was similar to the Beaufort scale, but with higher wind speeds for each category. These were recorded each hour as single force values, or a range, for example 2–3, or occasionally over several forces such as 2–4. We have used the mean force value for each hour, so a range of 2–3 is expressed as 2.5. Roy (2004) tabulates the conversion from force to knots, and Table 1 also shows m/s and mph. We fitted a fourth‐order polynomial to these thresholds and this was used to derive wind speeds for all forces, including interpolating to non‐integer values.

The calculation of relative humidity from the dry‐ and wet‐bulb temperatures, pressure and wind force observations is described and analysed in Burt and Hawkins (2019). Several examples of near‐zero relative humidity events at the summit are also described.

### 3.4 Data completeness and other data issues

The dataset produced has only a few gaps due to equipment failures when the original observations were made. A fraction of the values were published in square brackets indicating that they were estimates—these observations have been retained but are not flagged. One slightly odd feature of the data is the hourly precipitation at Fort William has a disproportionately large number of dry hours at 11 a.m. (evident from inspection of Figure 4). No satisfactory physical explanation is offered, and this is a potential observation bias.

## 4 DIGITIZED HOURLY WEATHER OBSERVATIONS

**Figure 3:**
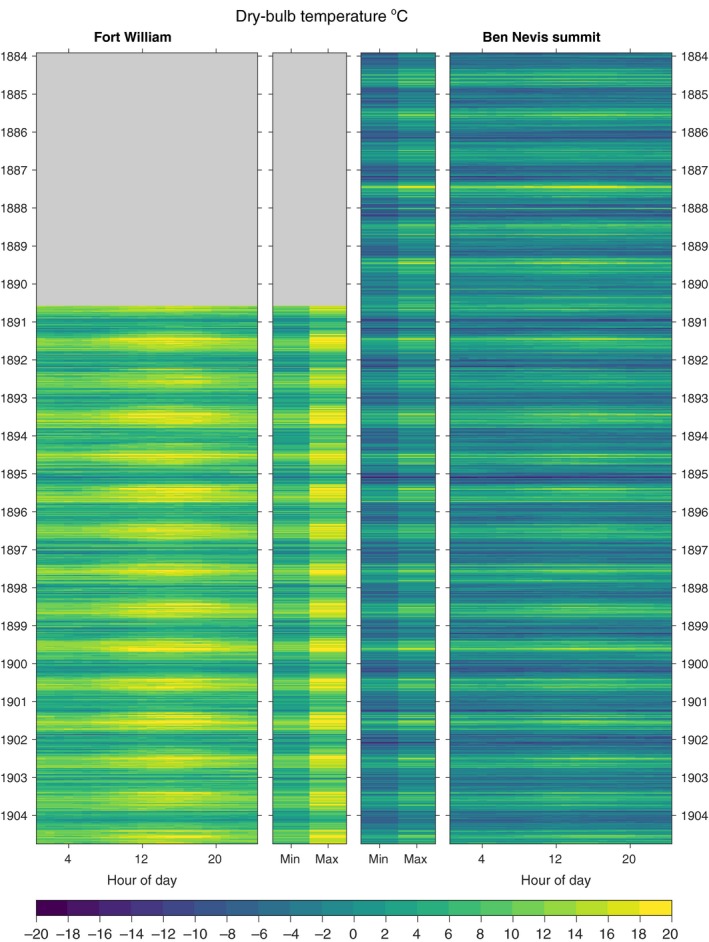
Dry‐bulb temperature observations for Ben Nevis and Fort William, showing the hourly and daily extreme data. Grey regions indicate data not available

**Figure 4:**
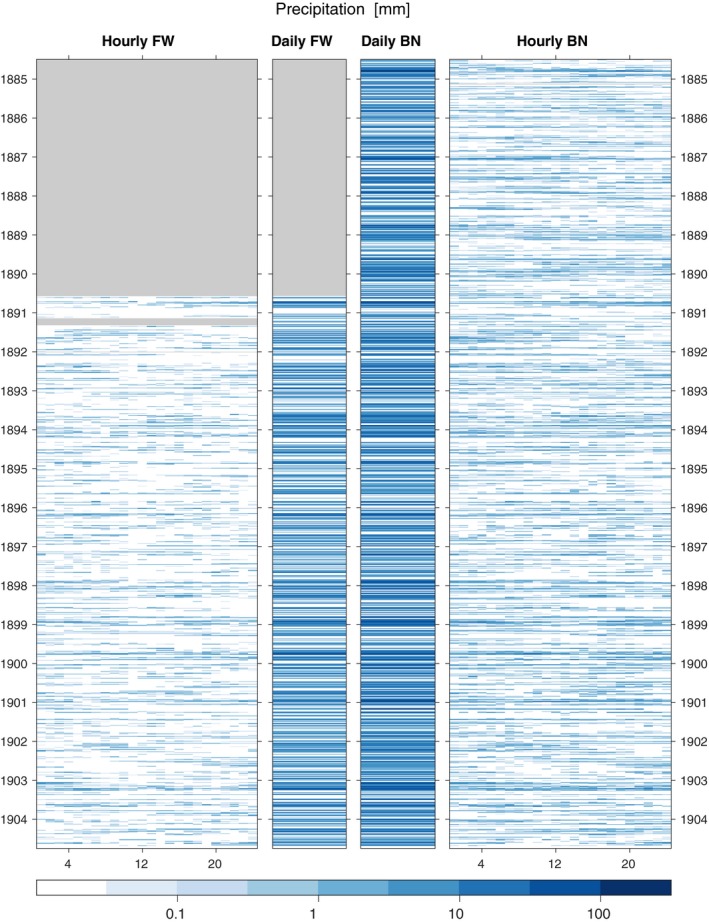
Precipitation observations for Ben Nevis (BN) and Fort William (FW), showing the hourly and daily data, in mm. Grey regions indicate data not available

**Figure 5:**
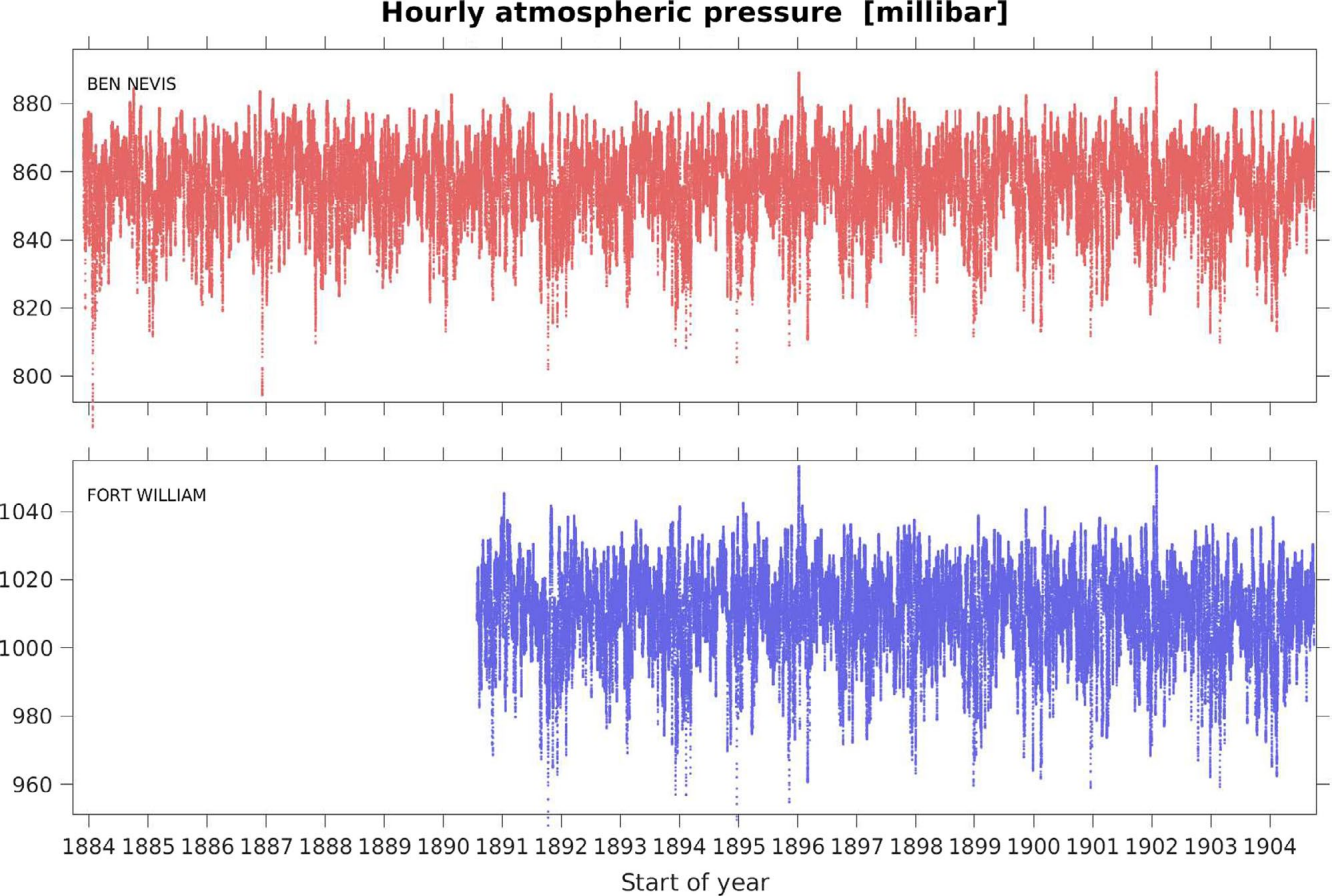
Hourly pressure values (corrected to 0°C) are in hPa (mb), and are station level pressure for Ben Nevis and reduced to mean sea level for Fort William

**Figure 6:**
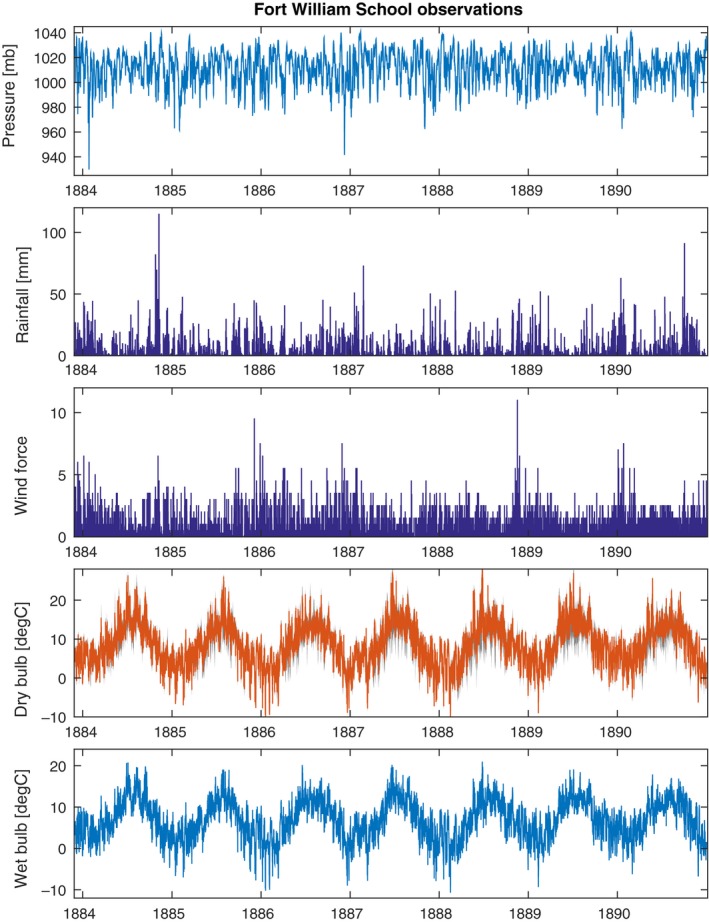
The observations taken at Fort William School. These are five times daily for temperature, sea level pressure and wind force (Beaufort Scale), and daily for rainfall. Cloud amount (not shown) is also available. The grey shading for the dry‐bulb temperature indicates the daily minimum to maximum range

The digitized data for dry‐bulb temperature, precipitation and pressure for both observatories are shown in Figures 3, 4, 5, respectively. The observations made at Fort William School are shown in Figure 6.

**Figure 7:**
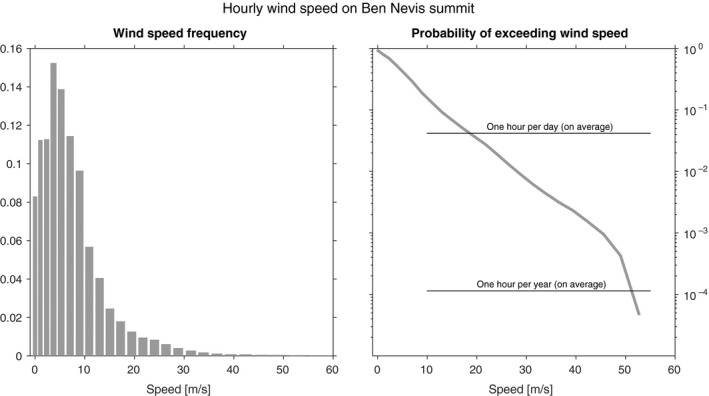
Wind speed frequency for Ben Nevis summit using hourly observations for 1893–1904, and the chance of exceeding a particular wind speed

For summit wind speed, we have produced a frequency histogram (Figure 7) to highlight the distribution of summit wind speeds, and the chance of exceeding a certain wind speed. Data exists for each hour on 4,290 days. Ben Nevis Force 12 (in excess of 113 kn, 57 m/s) was observed on only one occasion—between 0800 and 1400 GMT on 2 April 1901—and 15 days experienced Force 11 or higher—roughly once per year on average.

The winds observed at the summit are much affected by the local topography. For example, where the pressure pattern would have indicated very strong westerlies or north‐westerlies, the wind was deflected around the nearby peak of Carn Dearg and lighter than expected but very gusty northerlies were observed at the summit (Roy, 2004). This is likely to lead to an underestimation of the climatology relating to the strength of the wind on nearby summits.

### 4.1 Average and extreme values

For Ben Nevis summit, the mean annual temperature between 1884 and 1903 was −0.3°C. The lowest hourly temperatures recorded were −17.4°C (dry bulb) and −17.6°C (wet bulb) on Ben Nevis on 6 January 1894, and −11.4°C (dry bulb) and −11.7°C (wet bulb) in Fort William on 28 January 1895. The highest hourly temperatures were 19.1°C (dry bulb, 28 June 1902) and 14.4°C (wet bulb, 18 June 1893) on Ben Nevis, and 27.2°C (dry bulb, 9 August 1893) and 20.8°C (wet bulb, 6 September 1898) in Fort William. The coldest day on the summit occurred on 7 February 1895 when the average temperature was −16.0°C (minimum −16.8°C, maximum −15.0°C); the daily mean wind speed on that date was 18 m/s. Because the summit observatory was sited on a narrow plateau, the temperatures measured there were essentially those in the free air, whereas much lower temperatures are recorded in Scotland at inland low‐level sites under inversion conditions.

The lowest pressure observed at the summit of Ben Nevis was 784.9 mb on 26 January 1884, roughly equivalent to 929 mb at sea level. The highest pressure was 889.2 mb (or 1052 mb at sea level) on 31 January 1902. On the same day, the Fort William Observatory recorded 1053 mb and Aberdeen recorded 1053.6 mb—the highest pressure ever observed in the British Isles (Burt, 2007).

The most precipitation during a day at the summit observatory was 185 mm on 3 October 1890. On 10 December 1884, 33 mm fell in a single hour. The corresponding records at the Fort William Observatory were 79 and 16 mm, respectively, on different days. However, Fort William School recorded 115 mm on 8 November 1884.

### 4.2 Case study: February 1903

In late February 1903, a severe storm hit the British Isles, causing considerable damage to trees and buildings (Shaw, 1903), including 3,000 trees blown down in Phoenix Park, Dublin. This event is now known as the ‘Ulysses’ storm as these impacts were mentioned in the novel of the same name by James Joyce, with the events being set in 1904, the year after the storm (Joyce, 1922):

Lady Dudley was walking home through the park to see all the trees that were blown down by that cyclone last year and thought she’d buy a view of Dublin. (Joyce, 1922)

**Figure 8:**
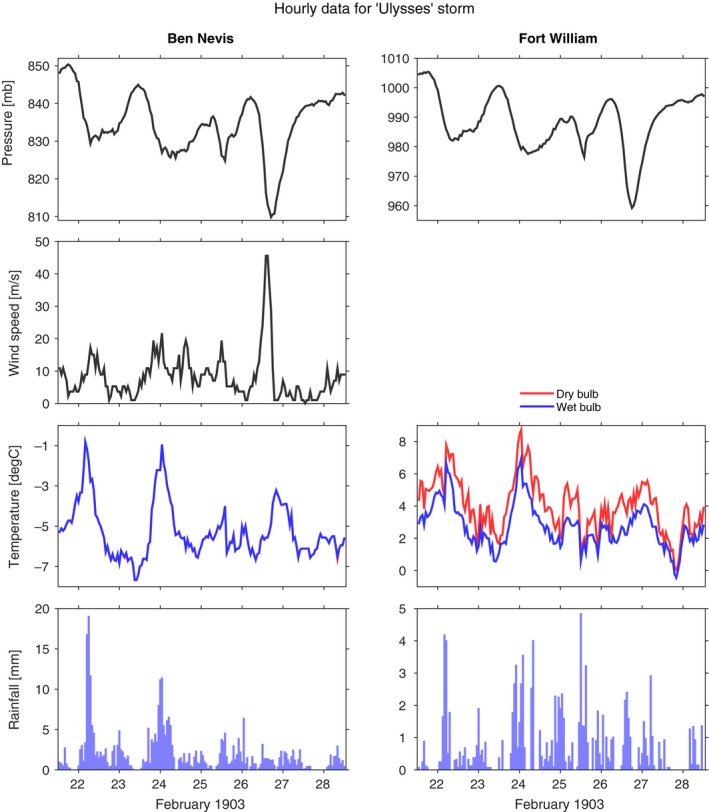
A severe storm hit the UK in late February 1903, known as the *Ulysses* storm. The Ben Nevis and Fort William observations provide a valuable account for understanding this particular storm, and similar extreme events. For Ben Nevis, the observed wet‐ and dry‐bulb temperatures are almost identical for the whole period shown, indicating very high humidity. For Ben Nevis, the pressure values are at station level (1345 m), and for Fort William, they are reduced to sea level. Note that the scales differ between the two observatories

The rescued hourly observations for this event are shown in Figure 8, showing the detail now available. For example, the wind speed can be seen to increase just before the storm passes over Ben Nevis, before dropping rapidly.

Assimilating these newly rescued pressures into long centennial reanalyses (e.g. Compo *et al*., 2011; Slivinski *et al*., 2019) will improve the dynamical reconstruction of this event and many similar storms and interesting weather events.

## 5 AURORAL OBSERVATIONS

Many modern technological systems are prone to disruption or damage from space weather phenomena (Baker, 2002), and cost‐effective design of these systems requires us to have an accurate climatology of near‐Earth space (Lockwood *et al.*, 2019). The problem in constructing such a climatology is that we have direct measurements of near‐Earth space from only the last 50 years which is inadequate to characterize the range of possible conditions, especially considering the dominant variation is the decadal‐scale sunspot cycle, added to which are centennial‐scale drifts.

To try to build a useful space climatology, historic ground‐based observations such as telescopic observations of sunspots (from 1612 onwards), magnetometer observations of geomagnetic activity (from about 1845 onwards) and naked‐eye observations of the aurora are used (Lockwood *et al.*, 2017). Potentially, the auroral data stretch back over millennia, but they are very inhomogeneous in reliability and subject to many climate and social factors and so there are major problems in interpreting them. As a consequence, auroral sightings have not been used as much.

Because of the offset of the geographic and geomagnetic poles, the geographic latitude of peak auroral occurrence varies with longitude, but the available hours of darkness and its seasonality depends on geographic latitude. Hence, the probability of observation varies with both latitude and longitude. Furthermore, the secular change in the geomagnetic field means that the consequent annual and diurnal variations in the probability of observing aurora depend not only on longitude but also on time. Added to the biases that this causes, there are other spatial and temporal factors such as the distribution of population, of cloud cover and of street lighting, and the willingness of a society to keep records of natural phenomena. All of these factors mean that global statistics on the occurrence of low‐latitude aurora do not form a homogeneous metric.

**Figure 9:**
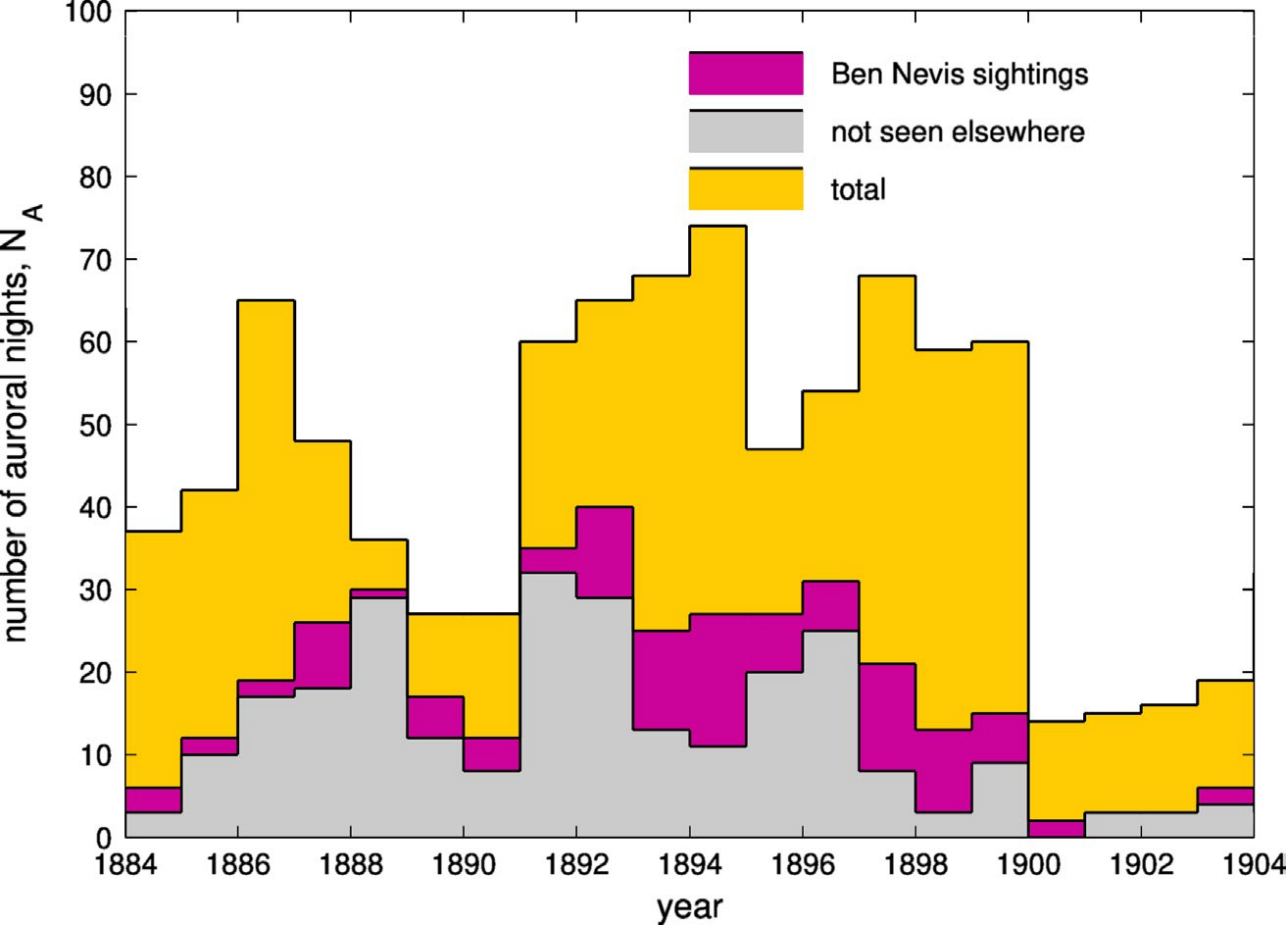
Number of UK auroral sightings on Ben Nevis (purple), and those not seen elsewhere in the UK (grey). The total number from all sources is shown by the yellow histogram

One way to reduce these problems is to restrict the longitudes used to compile the statistics, and for this reason, Lockwood and Barnard (2015) compiled a catalogue of sightings from the UK. After 1900, we have an excellent record of aurorae in the UK, with data collected from observatories and the many (manned) lighthouses that were constructed around Scottish coasts in the 19th century. As we extend the sequence before 1900, the record increasingly depends upon a few key regular observers and serendipitous observations reported in newspapers. But, in the late 19th century the Ben Nevis Observatory was a prime location for detecting aurorae. Figure 9 is an overview of the Ben Nevis observations, of which 374 were recorded. The mauve histogram shows the number of nights per year on which aurora was observed at the observatory and the grey histogram the number of nights where such observations were not matched by an observation on the same night at a different location in the UK. This points to a general under‐reporting of aurora at this time. The orange histogram shows the total number of nights on which aurora was seen in the UK. There were many nights on which aurora was seen elsewhere, but not at Ben Nevis, which also suggests that cloud cover there limited the number of auroral observations.

Data from observatories, or from experienced regular reporters of meteorological phenomena, have a major advantage over the opportunistic sightings, because it is known when it could not have been observed. The supporting information of cloud conditions at the observatory would be important in interpreting all the UK data as it will help us make a statistical allowance for the effect of cloud in studying the occurrence probability. However, these cloud observations have yet to be rescued.

## 6 CONCLUSIONS

Thousands of citizen scientists have successfully rescued millions of weather observations taken every hour at two nearby sites in the Scottish Highlands between 1883 and 1904. The use of volunteers allowed the digitization of the data to be achieved more quickly and more cheaply than commerical digitization. This project built on the success of http://OldWeather.org (Freeman *et al.*, 2017) and has since been adopted in a new phase of http://WeatherRescue.org, and by other projects such as http://SouthernWeatherDiscovery.org.

These observations will be passed to the Met Office to be included in the official UK weather records and to the Copernicus Climate Change Data Rescue Service to be added to the international databases. The Ben Nevis auroral observations also help fill in a gap in our auroral sightings records.

The data recorded so diligently over a century ago on top of a cold, wet, windy mountain are now available for anyone to analyse. The legacy of the dedicated observers will be a permanent record of the weather they experienced over a century ago.

### OPEN PRACTICES

This article has earned an Open Data badge for making publicly available the digitally‐shareable data necessary to reproduce the reported results. The data is available at https://catalogue.ceda.ac.uk/uuid/1d29816cee7e4fb586b80a3f7debcb8e. Learn more about the Open Practices badges from the Center for Open Science: https://osf.io/tvyxz/wiki.
